# Antitumor Activity of Gold(I), Silver(I) and Copper(I) Complexes Containing Chiral Tertiary Phosphines

**DOI:** 10.1155/MBD.1998.217

**Published:** 1998

**Authors:** Mark J. McKeage, Peter Papathanasiou, Geoffrey Salem, Allan Sjaarda, Gerhard F. Swiegers, Paul Waring, S. Bruce Wild

**Affiliations:** 1 Department of Pharmacology and Clinical Pharmacology Faculty of Medicine and Health Services The University of Auckland Private Bag 92019 Auckland New Zealand; 2 Australian National University Chemistry Department The Faculties A.C.T Canberra 0200 Australia; 3 Australian National University John Curtin School of Medical Research A.C.T Canberra 0200 Australia; 4 Australian National University Research School of Chemistry A.C.T Canberra 0200 Australia

## Abstract

The *in vitro* cytotoxicities of a number of gold(I), silver(I) and copper(I) complexes containing chiral tertiary phosphine ligands have been examined against the mouse tumour cell lines P815 mastocytoma, B16 melanoma [gold(I) and silver(I) compounds] and P388 leukaemia [gold(I) complexes only] with many of the complexes having IC_50_ values comparable to that of the reference compounds *cis*-diamminedichloroplatinum(ll), cisplatin, and bis[1,2-bis(diphenylphosphino) ethane]gold(I) iodide. The chiral tertiary phosphine ligands used in this study include (*R*)-(2-aminophenyl)methylphenylphosphine; (*R,R*)-, (*S,S*)- and (R^*^,R^*^)-1,2-phenylenebis(methylphenylphosphine); and (*R,R*)-, (*S,S*)- and (R^*^,R^*^)-bis{(2-diphenylphosphinoethyl)phenylphosphino}ethane. The *in vitro* cytotoxicities of gold(I) and silver(I) complexes containing the optically active forms of the tetra(tertiary phosphine) have also been examined against the human ovarian carcinoma cell lines 41M and CH1, and the cisplatin resistant 41McisR, CH1cisR and SKOV-3 tumour models. IC_50_ values in the range 0.01 - 0.04 μM were determined for the most active compounds, silver(I) complexes of the tetra(tertiary phosphine). Furthermore, the chirality of the ligand appeared to have little effect on the overall activity of the complexes: similar IC_50_ data were obtained for complexes of a particular metal ion with each of the stereoisomeric forms of a specific ligand.


### ANTITUMOR ACTIVITY OF GOLD(I), SILVER(I) AND COPPER(I) COMPLEXES CONTAINING CHIRAL TERTIARY PHOSPHINES

Mark J. McKeage,[1] Peter Papathanasiou,[2a] Geoffrey Salem*,[2a] Allan Sjaarda,[2b] Gerhard F. Swiegers,[2c] Paul Waring[2b] and S. Bruce Wild[2c]

[1] Department of Pharmacology and Clinical Pharmacology, Faculty of Medicine and Health Services, The University of Auckland, Private Bag 92019, Auckland, New Zealand
[2] Australian National University, Canberra, A.C.T. 0200, Australia
[a] Chemistry Department, The Faculties, [b] John Curtin School of Medical Research, [c] Research School of Chemistry

**Abstract.**
The *in vitro* cytotoxicities of a number of gold(I), silver(I) and copper(I) complexes containing chiral tertiary phosphine ligands have been examined against the mouse tumour cell lines P815 mastocytoma, B16 melanoma [gold(I) and silver(I) compounds] and P388 leukaemia [gold(I) complexes only] with many of the complexes having $IC_{50}$ values comparable to that of the reference compounds *cis*-diamminedichloroplatinum(II), cisplatin, and bis[1,2-bis(diphenylphosphino)ethane]gold(I) iodide. The chiral tertiary phosphine ligands used in this study include (*R*)-(2-aminophenyl)methylphenylphosphine; (*R*,*R*)-, (*S*,*S*)- and (*R**,*R**)-1,2-phenylenebis(methylphenylphosphine); and (*R*,*R*)-, (*S*,*S*)- and (*R**,*R**)-bis{(2-diphenylphosphinoethyl)phenylphosphino}ethane. The *in vitro* cytotoxicities of gold(I) and silver(I) complexes containing the optically active forms of the tetra(tertiary phosphine) have also been examined against the human ovarian carcinoma cell lines 41M and CH1, and the cisplatin resistant 41McisR, CH1cisR and SKOV-3 tumour models. $IC_{50}$ values in the range 0.01 - 0.04 μM were determined for the most active compounds, silver(I) complexes of the tetra(tertiary phosphine). Furthermore, the chirality of the ligand appeared to have little effect on the overall activity of the complexes: similar $IC_{50}$ data were obtained for complexes of a particular metal ion with each of the stereoisomeric forms of a specific ligand.

**Introduction.**
Bis(ditertiary phosphine) complexes of gold(I), silver(I) and copper(I) have attracted much interest over the past decade as certain of these complexes have been shown to display antitumour activities comparable to *cis*-diamminedichloroplatinum(II), cisplatin, for example, bis[1,2-bis(diphenylphosphino)ethane]gold(I) chloride, $[Au(dppe)_2]Cl$, and its silver(I) and copper(I) derivatives.[1] The mode of action of these complexes is not known but it is believed to be quite different to that of cisplatin. The complex $[Au(dppe)_2]Cl$ has been shown to inhibit DNA, RNA and protein synthesis; to cause DNA single strand breaks; and to induce mitochondrial damage in hepatocytes. Indeed, despite the early promise of $[Au(dppe)_2]Cl$ and its derivatives, the compounds have subsequently been shown to be too toxic for clinical use due to side-effects related to the disruption of mitochondrial function in hepatocytes.[2,3]

We have recently synthesised a number of related gold(I), silver(I) and copper(I) complexes containing the substituted (2-aminophenyl)phosphines AMPP [(±)-(2-aminophenyl)methylphenylphosphine] and ADPP [(2-aminophenyl)diphenylphosphine] and investigated their *in vitro* cytotoxic properties against three mouse tumour cell lines, P815 mastocytoma, B16 melanoma and P388 leukaemia.[4] Certain of these complexes exhibited antiproliferative properties comparable to those of the two reference compounds cisplatin and $[Au(dppe)_2]I$. Furthermore, the complexes $[Au(ADPP)_2]PF_6$ and $[Au\{(\pm)\text{-}AMPP\}_2]PF_6$ were shown to rapidly accumulate in mitochondria by virtue of their lipophilic, cationic nature and to inhibit the activity of ATP synthase leading to rapid cell death.[5]

In this paper we report on the *in vitro* cytotoxic properties of a range of gold(I), silver(I) and copper(I) complexes containing chiral tertiary arsine and phosphine ligands against the P815 tumour cell line. $IC_{50}$ values have also been determined for certain of these complexes against the tumour cell lines B16, P388, 41M, and CH1, and the cisplatin resistant 41McisR, CH1cisR and

SKOV-3 tumour models. While the chirality of platinum-based antitumour agents is known to have a dramatic influence on their activity,[6] this effect has not previously been investigated for gold(I), silver(I) or copper(I) complexes containing tertiary phosphine ligands.

**Materials and Methods.**

(a) *Syntheses.* The ligands (±)-(2-aminophenyl)methylphenylphosphine,[7] (*R**,*R**)-1,2-phenylenebis(methylphenylphosphine)[8] and (*R**,*R**)-1,2-bis{(2-diphenylphosphinoethyl)phenylphosphino}-ethane[9] were synthesised and resolved following literature procedures. The complexes containing (*R*,*R*)-, (*S*,*S*)- and (*R**,*R**)-dias or -diph [M = Ag(I) or Cu(I)][10] and (*R*,*R*)- and (*S*,*S*)-tetraphos [M = Au(I), Ag(I) or Cu(I)][9] were prepared as previously described in the literature. The synthesis of $[Au_2\{(R^*,R^*)\text{-tetraphos}\}_2](PF_6)_2$, $[Au_2\{(R^*,S^*)\text{-tetraphos}\}_2](PF_6)_2$, $[Ag_2\{(R^*,R^*)\text{-tetraphos}\}_2]\text{-}(PF_6)_2$, and $[Cu\{(R^*,R^*)\text{-tetraphos}\}]PF_6$ will be reported elsewhere.[11]

*Synthesis of [L-2]-Bis[(R)-(2-aminophenyl)methylphenylphosphine]gold(I) Iodide Ethanol Solvate, [Au{(R)-AMPP}₂]I.* The complex was prepared in a similar manner to that described for $[Au\{(\pm)\text{-}AMPP\}_2]I$.[4] A solution of the optically active ligand (*R*)-AMPP (0.17 g, 0.79 mmol) in ethanol (5 mL) was added to a solution of tetra-*n*-butylammonium diiodoaurate(I) (0.27 g, 0.39 mmol) in ethanol (15 mL) with stirring. The white crystalline product was filtered off, washed with ethanol and dried *in vacuo* (0.24 g, 83%), m.p. 172 °C. (Found: C, 42.3; H, 4.2; N, 3.3. Calc. for $C_{28}H_{34}AuIN_2OP_2$: C, 42.0; H, 4.3; N, 3.5%). $^1H$ NMR ($CDCl_3$): δ 2.26 (d, 6 H, $^2J_{PH}$ 8.5 Hz, PMe), 4.31 (s, 4 H, $NH_2$), 6.63-7.67 (m, 18 H, aromatics). $^{31}P\text{-}\{^1H\}$ NMR ($CDCl_3$): δ 11.1 (s, 2 P). $\Lambda_M$ ($MeNO_2$): 92 $cm^2$ S $mol^{-1}$; $\Lambda_M$ (MeCN): 92 $cm^2$ S $mol^{-1}$.

*Synthesis of [T-4]-Bis[(R*,R*)-1,2-phenylenebis(methylphenylphosphino)benzene]gold(I) Iodide, [Au{(R*,R*)-diph}₂]I.* Tetra-*n*-butylammonium diiodoaurate(I) (0.22 g, 0.32 mmol) was dissolved in ethanol (20 mL) and the ligand (*R**,*R**)-diph (0.20 g, 0.62 mmol) was slowly added with stirring. The solution was stirred for 30 min and then the solvent was removed by evaporation. The residue was dissolved in acetone (5 mL) and diethyl ether was added dropwise to give a white crystalline product which was filtered off, washed with diethyl ether and dried *in vacuo* (0.24 g, 80%), m.p. 160 °C (Found: C, 50.0; H, 4.3. Calc. for $C_{40}H_{40}AuIP_4$: C, 49.6; H, 4.2%). $^1H$ NMR ($CDCl_3$): δ 2.27 (s, 12 H, PMe), 7.12-7.53 (m, 28 H, aromatics). $^{31}P\text{-}\{^1H\}$ NMR ($CDCl_3$): δ 4.6 (s, 4 P). $\Lambda_M$ ($MeNO_2$): 93 $cm^2$ S $mol^{-1}$; $\Lambda_M$ (MeCN): 94 $cm^2$ S $mol^{-1}$.

*Synthesis of [T-4]-Bis[(R,R)-1,2-phenylenebis(methylphenylphosphino)benzene]gold(I) Iodide, [Au{(R,R)-diph}₂]I.* Prepared as for $[Au\{(R^*,R^*)\text{-diph}\}_2]I$ except using tetra-*n*-butylammonium diiodoaurate(I) (0.16 g, 0.23 mmol) and the ligand (*R*,*R*)-diph (0.15 g, 0.47 mmol). The product was recrystallised from acetone (3 mL) by the dropwise addition of diethyl ether to give white crystals (0.18 g, 82%), m.p. 160 °C. (Found: C, 49.7; H, 4.3. Calc. for $C_{40}H_{40}AuIP_4$: C, 49.6; H, 4.2%). $^1H$ NMR ($CDCl_3$) and $^{31}P\text{-}\{^1H\}$ NMR ($CDCl_3$): identical to that recorded for $[Au\{(R^*,R^*)\text{-diph}\}_2]I$. $\Lambda_M$ ($MeNO_2$): 93 $cm^2$ S $mol^{-1}$; $\Lambda_M$ (MeCN): 94 $cm^2$ S $mol^{-1}$.

*Synthesis of [T-4]-Bis[(S,S)-1,2-phenylenebis(methylphenylphosphino)benzene]gold(I) Iodide, [Au{(S,S)-diph}₂]I.* Prepared as for $[Au\{(R^*,R^*)\text{-diph}\}_2]I$ except using tetra-*n*-butylammonium diiodoaurate(I) (0.16 g, 0.23 mmol) and the ligand (*S*,*S*)-diph (0.15 g, 0.47 mmol). The product was obtained as white crystals after recrystallisation from acetone - diethyl ether (0.21 g, 94%), m.p. 160 °C. (Found: C, 49.3; H, 4.1. Calc. for $C_{40}H_{40}AuIP_4$: C, 49.6; H, 4.2%). $^1H$ NMR ($CDCl_3$) and $^{31}P\text{-}\{^1H\}$ NMR ($CDCl_3$): identical to that recorded for $[Au\{(R^*,R^*)\text{-diph}\}_2]I$. $\Lambda_M$ ($MeNO_2$): 93 $cm^2$ S $mol^{-1}$; $\Lambda_M$ (MeCN): 94 $cm^2$ S $mol^{-1}$.

*Synthesis of [T-4]-Tris[(R)-(2-aminophenyl)methylphenylphosphine]silver(I) Hexafluorophosphate, [Ag{(R)-AMPP}₃]PF₆.* The complex was prepared in a similar manner to that described for $[Ag\{(\pm)\text{-}AMPP\}_3]PF_6$.[4] A solution of (*R*)-AMPP (0.09 g, 0.42 mmol) in ethanol (5 mL) was added dropwise to a solution of silver nitrate (0.02 g, 0.12 mmol) in ethanol (25 mL). The solution was filtered, the solvent removed by evaporation, the residue redissolved in dichloromethane (10 mL) and a solution of $NH_4PF_6$ (0.20 g, 1.22 mmol) in water (5 mL) added. The organic layer was separated off, dried over anhydrous $MgSO_4$, filtered and the solvent again removed by evaporation. The residue was redissolved in methanol (5 mL) and gave a white crystalline product on standing. The product was collected, washed with diethyl ether (5 mL), dried *in vacuo* and stored in the dark (0.09 g, 82%), m.p. 168 °C. (Found: C, 52.0; H, 4.4; N, 4.5. Calc. for $C_{39}H_{42}AgF_6N_3P_4$: C, 52.1; H, 4.7; N, 4.7%). $^1H$ NMR ($CDCl_3$): δ 1.82 (br s, 9 H, PMe), 4.75 (s, 6 H, $NH_2$), 6.80-7.54 (m, 27 H, aromatics). $^{31}P\text{-}\{^1H\}$ NMR ($CDCl_3$): δ -22.5 (s, 3 P). $\Lambda_M$ ($MeNO_2$): 83 $cm^2$ S $mol^{-1}$; $\Lambda_M$ (MeCN): 84 $cm^2$ S $mol^{-1}$.

(b) *Biological procedures and materials. (i) Mouse tumour cell lines*: P815 mastocytoma cells were cultured in Eagle's Minimum Essential Medium F15 with 10% foetal bovine serum; B16 cells in McCoy's medium RPMI 1640 with 10% foetal bovine serum; and P388 leukaemia cells in Dulbecco's Modified Eagle Medium H16 with 10% horse serum (herein EC10). All cells were cultured and incubated in a Forma Scientific Infrared $CO_2$ incubator at 37 °C in 5% $CO_2$. Cell suspensions were centrifuged using a Jouan centrifuge model CR 4 22. Linbro 96 round bottom well tissue culture plates were used for the thymidine incorporation assay. Cells were harvested using a Pharmacia Version 1.02 Micro Cell Harvester and incorporated thymidine was counted using a Pharmacia 1205 Betaplate liquid scintillation counter. Thymidine incorporation assays were performed following a literature procedure.[12] The compounds tested were dissolved in dimethylsulfoxide (0.2 mL) to give a concentration of 0.02 M and then diluted to $2 \times 10^{-5}$ M using EC10.

(ii) *Human ovarian carcinoma cell lines*: The biological properties of the CH1, 41M and SKOV-3 cell lines have been described previously.[13,14] The cell-lines were grown as monolayers in Dulbecco's modified Eagle plus10% foetal calf serum, 100 μg $mL^{-1}$ streptomycin, 100 μg $mL^{-1}$ penicillin and 2 mM glutamine in a 5% $CO_2$ atmosphere. The cell lines were checked regularly for mycoplasma infection. Cytotoxicity assessment was undertaken using the sulphorhodamine B assay as described previously. [14] The complexes were dissolved in dimethylsulfoxide, diluted in distilled water and added to the cultures at concentrations ranging from 0.005 to 5 μM in quadruplicate. The final concentration of dimethylsulfoxide in the culture medium was < 0.5%. The cells were exposed to the drug containing medium for 96 h. The $IC_{50}$ values were determined as the drug concentration reducing absorption (564 nm) of sulphorhodamine B-stained wells to 50% of that of the control wells.

**Results.**

*Complexes.* A number of univalent gold, silver and copper complexes have been utilised in the present study containing the ligands (*R*)-(2-aminophenyl)methylphenylphosphine (AMPP); (*R*,*R*)-, (*S*,*S*)- and (*R**,*R**)-1,2-phenylenebis(methylphenylphosphine) (diph) and their arsenic analogues (dias); and (*R*,*R*)-, (*S*,*S*)-, (*R**,*R**)- and (*R**,*S**)-bis{(2-diphenylphosphinoethyl)phenylphosphino}-ethane (tetraphos).†

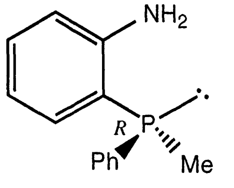

AMPP

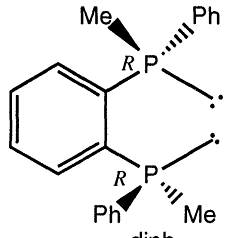

diph

(Only one enantiomer of each ligand is shown.)

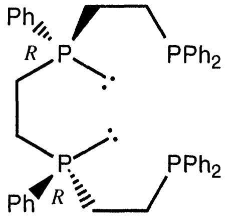

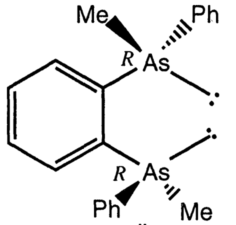

tetraphos dias

The complexes that were used in this work are listed in Tables 1-4. We have previously described the preparation and characterisation of many of these complexes.[9,10] Complexes containing (*R*)-AMPP were prepared in a similar fashion to that reported for their racemic analogues.[4] Gold(I) compounds of diph were synthesised by reacting two equivalents of the appropriate form of the ligand with $NBu_4[AuI_2]$. We have previously prepared the corresponding hexafluorophosphate salts via an alternate route.[15]

† No gold(I) complexes of dias; silver(I) compounds of (*R*,*R*)-dias, (*R*,*R*)-diph and (*R**,*S**)-tetraphos; and copper(I) complexes of (*R*)-AMPP, (*R**,*S**)-tetraphos and (*S*,*S*)-diph were studied in this work.

*Biological Studies.* The *in vitro* cytotoxic properties of the gold(I), silver(I) and copper(I) complexes have been assessed by measuring their effect on proliferation of the mouse tumour cell lines P815 mastocytoma, B16 melanoma [gold(I) and silver(I) compounds] and P388 leukaemia [gold(I) complexes only].

**Table 1.** $IC_{50}$ values for gold(I) complexes and cisplatin against P815 mastocytoma, B16 melanoma, and P388 leukaemia tumour models

| Complex | $IC_{50}$ (μM) P815 | B16 | P388 |
|---|---|---|---|
| [Au{(±)-AMPP}$_2$]I[4] | 9.25 | 2.60 | 5.80 |
| [Au{(*R*)-AMPP}$_2$]I | 9.00 | 3.00 | 5.50 |
| [Au{(*R**,*R**)-diph}$_2$]I | 0.75 | 5.00 | 0.20 |
| [Au{(*R*,*R*)-diph}$_2$]I | 0.90 | 4.70 | 1.10 |
| [Au{(*S*,*S*)-diph}$_2$]I | 2.45 | 3.50 | 2.30 |
| [Au$_2${(*R**,*R**)-tetraphos}$_2$](PF$_6$)$_2$ | 0.87 | 0.55 | 0.60 |
| [Au(dppe)$_2$]I[4] | 0.22 | 5.20 | 0.10 |
| *cis*-[PtCl$_2$(NH$_3$)$_2$][4] | 14.50 | 0.90[1] | 5.00 |

**Table 2.** $IC_{50}$ values for silver(I) complexes against P815 mastocytoma and B16 melanoma mouse tumour cell models

| Complex | $IC_{50}$ (μM) P815 | B16 |
|---|---|---|
| [Ag{(±)-AMPP}$_3$]PF$_6$[4] | 9.50 | 5.00 |
| [Ag{(*R*)-AMPP}$_3$]PF$_6$ | 9.25 | 5.00 |
| [Ag{(*R**,*R**)-diph}$_2$]PF$_6$ | 0.14 | 4.00 |
| [Ag{(*S*,*S*)-diph}$_2$]PF$_6$ | 1.62 | 4.30 |
| [Ag{(*R**,*R**)-dias}$_2$]PF$_6$ | 0.75 | 1.30 |
| [Ag{(*S*,*S*)-dias}$_2$]PF$_6$ | 0.32 | 1.40 |
| [Ag$_2${(*R**,*R**)-tetraphos}$_2$](PF$_6$)$_2$ | 0.04 | - |

**Table 3.** $IC_{50}$ values for copper(I) complexes and the free ligands against P815 mastocytoma mouse tumour cell model

| Complex | $IC_{50}$ (μM) |
|---|---|
| [Cu{(±)-AMPP}$_2$]PF$_6$[4] | 49.00 |
| [Cu{(±)-AMPP}$_3$]PF$_6$[4] | 6.00 |
| [Cu{(*R**,*R**)-diph}$_2$]PF$_6$ | 0.14 |
| [Cu{(*R*,*R*)-diph}$_2$]PF$_6$ | 1.45 |
| [Cu{(*R**,*R**)-dias}$_2$]PF$_6$ | 0.57 |
| [Cu{(*R*,*R*)-dias}$_2$]PF$_6$ | 0.52 |
| [Cu{(*S*,*S*)-dias}$_2$]PF$_6$ | 0.35 |
| [Cu{(*R**,*R**)-tetraphos}]PF$_6$ | 0.15 |
| [Cu{(*R*,*R*)-tetraphos}]PF$_6$ | 0.16 |
| [Cu{(*S*,*S*)-tetraphos}]PF$_6$ | 0.14 |
| **Ligand** | |
| (*R**,*R**)-diph | 0.30 |
| (*R*,*R*)-diph | 0.27 |
| (*S*,*S*)-diph | 0.49 |
| (*S*,*S*)-tetraphos | 0.85 |

The $IC_{50}$ values (concentrations resulting in 50% inhibition of labelled thymidine) for these complexes and the reference compounds cisplatin and $[Au(dppe)_2]I$ are given in Tables 1-3. $IC_{50}$ values for the ligands (*R**,*R**)-, (*R*,*R*)- and (*S*,*S*)-diph, and (*S*,*S*)-tetraphos against the P815 mastocytoma cell line are also included in Table 3. The *in vitro* cytotoxicities of gold(I) and silver(I) complexes containing the optically active or *meso* [for Au(I) only] forms of the tetra(tertiary phosphine) tetraphos have also been examined against the human ovarian carcinoma cell lines 41M and CH1, the acquired cisplatin resistant 41McisR and CH1cisR tumour models, and the intrinsically cisplatin resistant SKOV-3 line. $IC_{50}$ values (determined via a sulphorhodamine B assay) for these complexes are given in Table 4.

**Table 4.** $IC_{50}$ values for gold(I) and silver(I) complexes containing (*R*,*R*)-, (*S*,*S*)-, and (*R**,*S**)-tetraphos against P815, 41M, 41McisR, SKOV-3, CH1 and CH1cisR tumour cell lines

| Complex | $IC_{50}$ (μM) P815 | 41M | 41McisR | SKOV-3 | CH1 | CH1cisR |
|---|---|---|---|---|---|---|
| $[Au_2\{(R,R)\text{-tetraphos}\}_2](PF_6)_2$ | 0.82 | 0.42 | 0.55 | 0.47 | 0.40 | 0.20 |
| $[Au_2\{(S,S)\text{-tetraphos}\}_2](PF_6)_2$ | 0.95 | 0.39 | 0.43 | 0.45 | 0.34 | 0.16 |
| $[Au_2\{(R^*,S^*)\text{-tetraphos}\}_2](PF_6)_2$ | - | 0.35 | 0.47 | 0.30 | 0.31 | 0.15 |
| $[Ag_2\{(R,R)\text{-tetraphos}\}_2](PF_6)_2$ | 0.03 | 0.029 | 0.021 | 0.052 | 0.016 | 0.015 |
| $[Ag_2\{(S,S)\text{-tetraphos}\}_2](PF_6)_2$ | 0.02 | 0.029 | 0.022 | 0.077 | 0.016 | 0.012 |

**Discussion.**

*Nature of Complexes*. The structures of certain of the complexes used in this work have been confirmed by X-ray analysis.[4,9] Apart from the gold(I) complex of (*R*)-AMPP, *viz.* $[Au\{(R)\text{-AMPP}\}_2]I$, which is believed to have a linear geometry about the metal centre with the ligands bound in a monodentate fashion *via* the phosphorus donor atom,[4] in all other cases a tetrahedral stereochemistry prevails. The complex $[Ag\{(R)\text{-AMPP}\}_3]PF_6$ is believed to contain one bidentate ligand and two ligands bound in a monodentate fashion *via* the phosphorus donor atom. A similar structure has been proposed for related complexes of the type $[ML_3]PF_6$ [where M = Cu(I) or Ag(I); and L = (2-aminophenyl)diphenylphosphine, ADPP, or (±)-AMPP].[4] Gold(I) and silver(I) complexes of tetraphos are dimeric in the solid state, they form bimetallic helicates having double-helical or side-by-side helical structures.[9]

All of the complexes containing (*R*)-AMPP, dias or diph undergo rapid ligand exchange reactions in solution with the gold(I) compounds of the latter being the least kinetically labile.[4,10] Ligand exchange reactions for complexes containing the tetra(tertiary phosphine) appear to be much slower, for example, no exchange between the gold(I) complexes and added free ligand in $d^6$-DMSO was observed in the $^{31}P\text{-}\{^1H\}$ NMR spectra. Molar conductance measurements on gold(I), silver(I) and copper(I) complexes of tetraphos in acetonitrile, however, indicate that an equilibrium between monomeric and dimeric forms probably exists in solution.[9]

*Biological Studies*. As previously noted,[1,4] the nature of the metal ion and the counterion made very little difference to the cytotoxicity of the complexes. The silver(I) complexes of tetraphos were the exception. They exhibited significantly higher activities towards the P815 melanoma cell line than their gold(I) and copper(I) counterparts. This trend is not confined to one cell line but is clearly apparent in all of the cell lines tested including the cisplatin resistant models 41McisR, CH1cisR and SKOV-3. $IC_{50}$ values in the range 0.01 - 0.04 μM were determined for the silver(I) complexes of tetraphos. They are the most active of the compounds utilised in the present study.

Furthermore, the cytotoxicities of many of the complexes are comparable to that of *cis*-diamminedichloroplatinum(II), *cis*-$[PtCl_2(NH_3)_2]$, and bis[1,2-bis(diphenylphosphino)ethane]gold(I) iodide, $[Au(dppe)_2]I$. Those containing the quadridentate tetra(tertiary phosphine) tetraphos were generally found to be more active than those incorporating the bidentate di(tertiary phosphines) diph and dppe, which in turn exhibited greater cytotoxicities than compounds with AMPP ligands. A similar trend is generally observed with respect to the kinetic stabilities of these complexes. The data, however, do not reflect differences in kinetic stability based on the nature of the metal ion. The free tertiary phosphines themselves are also cytotoxic but generally to a significantly lesser extent than their metal complexes.

Complexes containing tertiary arsines are also active. The copper(I) and silver(I) compounds containing dias had a similar effect on the proliferation of P815 melanoma cells as their diph counterparts. The complexes of dias are significantly more labile than their diph analogues.[10]

The chirality of the ligand had no apparent effect on the cytotoxicity of the complex. In most cases similar $IC_{50}$ data were recorded irrespective of the isomeric form of the ligand coordinated to the metal ion. A ten-fold difference in activity was observed between $[Ag\{(R^*,R^*)\text{-diph}\}_2]PF_6$ and $[Ag\{(S,S)\text{-diph}\}_2]PF_6$ against the P815 but not the B16 cell lines. This data supports the notion that the biological target of these complexes is different to that of cisplatin. In the case of chiral platinum-based complexes a significant difference in activity has been observed between the two enantiomers; those containing the (*R*,*R*) antipode of a range of optically active diamines were more cytotoxic than either the (*S*,*S*) or racemic forms of the corresponding ligands.[6]

The $IC_{50}$ values reported in this work were typically determined over a period of 22 h: treated samples were incubated for 18 h prior to the addition of $^3$H-thymidine and then further incubated for 4 h. The data reported in Table 4 (except against P815) was determined over a period of 96 h using a sulphorhodamine B assay. As we reported previously for gold(I) complexes containing (±)-AMPP, (*R**,*R**)-tetraphos, dppe or ADPP;[5] a different reaction profile was observed when the $^3$H-thymidine incorporation assay was performed using the minimum time requirement for the experiment, i.e. $^3$H-thymidine was added after 0.25 h to the treated samples and then incubated for a further 0.5 h. No significant inhibition of DNA synthesis in the murine P388 leukaemia cell line was observed for cisplatin or $[Au_2(\text{tetraphos})_2]^{2+}$ under these conditions. Complete inhibition of DNA synthesis takes ~ 20 h at a concentration of 10 μM and 100 μM for $[Au_2(\text{tetraphos})_2]^{2+}$ and cisplatin, respectively. On the other hand, rapid inhibition of DNA synthesis was observed for gold(I) complexes containing AMPP, dppe or ADPP and furthermore their dose response did not substantially change over 24 h indicating that their effect was rapid and complete within 1 h. These complexes were also shown to cause a rapid increase in the mitochondrial membrane potential in intact P388 leukaemia cells which correlates with their early effect on DNA synthesis. The gold(I) complex of (*R**,*R**)-tetraphos, but not cisplatin, was also shown to cause an increase in the mitochondrial membrane potential when the experiment was performed over a period of 4 h. This mitochondrial activity has been shown to arise from inhibition of ATP synthase by the gold(I) complexes.[5]

The $IC_{50}$ data reported here further corroborate these earlier findings i.e. the primary target of gold(I), silver(I) and copper(I) complexes containing tertiary phosphine ligands is not DNA. A much more dramatic difference in $IC_{50}$ values would have been expected for complexes containing different stereoisomeric forms of the same ligand, if this was the case. Rather these gold(I) complexes [and presumably their silver(I) and copper(I) analogues] act by rapid accumulation in mitochondria by virtue of their lipophilic cationic nature followed by inhibition of the ATP synthase which ultimately results in the observed inhibition of cellular proliferation.

**References**

(1) S. J. Berners-Price and P. J. Sadler, *Struct. Bonding (Berlin)*, 1988, **70**, 27.
(2) G. D. Hoke, G. F. Rush, G. E. Bossard, J. V. McArdle, B. D. Jensen and C. K. Mirabelli, *J. Biol. Chem.*, 1988, **263**, 11203.
(3) P. F. Smith, G. D. Hoke, D. W. Alberts, P. J. Bugelski, S. Lupo, C. K. Mirabelli and G. F. Rush, *J. Pharmacol. Exp. Ther.*, 1989, **249**, 944.
(4) P. Papathanasiou, G. Salem, P. Waring and A. C. Willis, *J. Chem. Soc., Dalton Trans.*, 1997, 3435.
(5) P. Waring, A. Sjaarda, P. Papathanasiou, S. B. Wild and G. Salem, manuscript submitted to Cancer Research.
(6) T. W. Hambley, *Coord. Chem. Rev.*, 1997, **166**, 181.
(7) C. E. Barclay, G. Deeble, R. J. Doyle, S. A. Elix, G. Salem, T. L. Jones, S. B. Wild and A. C. Willis, *J. Chem. Soc., Dalton Trans.*, 1995, 57.

(8) N. K. Roberts and S. B. Wild, *J. Am. Chem. Soc.*, 1979, **101**, 6254.
(9) A. L. Airey, G. F. Swiegers, A. C. Willis and S. B. Wild, *Inorg. Chem.*, 1997, **36**, 1588.
(10) G. Salem, A. Schier and S. B. Wild, *Inorg. Chem.*, 1988, **27**, 3029.
(11) A. L. Airey, G. F. Swiegers and S. B. Wild, unpublished work.
(12) L. Hudson and F. C. Hay, *Practical Immunology*, 3rd ed., Blackwell Scientific Publications, London, 1989, p 430.
(13) C. A. Hills, L. R. Kelland, G. Abel, J. Siracky, A. P. Wilson and K. R. Harrap, *Br. J. Cancer*, 1989, **59**, 527.
(14) L. R. Kelland, P. Mistry, G. Abel, S. Y. Loh, C. F. O'Neill, B. A. Murrer and K. R. Harrap, *Cancer Res.*, 1992, **52**, 3857.
(15) J. A. L. Palmer and S. B. Wild, *Inorg. Chem.*, 1983, **22**, 4054.

**Received: July 1, 1998 - Accepted: July 14, 1998**

